# Genome Sequence of a Human Coxsackievirus A6 Strain Isolated from a Severe Hand, Foot, and Mouth Disease Case in Qingdao, China, in 2017

**DOI:** 10.1128/MRA.01449-19

**Published:** 2020-04-23

**Authors:** Zhilei Su, Xiaoyan Shi, Feng Zhang, Qing Chai, Jinling Gong, Zhaoguo Wang

**Affiliations:** a School of Public Health, Qingdao University, Qingdao, China; b Microbiological Laboratory, Qingdao Center for Disease Control and Prevention, Qingdao, China; Portland State University

## Abstract

In the present study, we describe the genome sequence of coxsackievirus A6 (CVA6) strain 17ES4/QD/CHN/2017, which was isolated in Qingdao, China, in 2017. According to the phylogenetic analyses, the isolate belongs to subgenotype D3a.

## ANNOUNCEMENT

Hand, foot, and mouth disease (HFMD) is a common infectious disease that mostly affects children less than 5 years of age; it is caused mainly by *Enterovirus* species A (1). Enterovirus A71 and coxsackievirus A16 (CVA16) are the two historically well-known serotypes. CVA6, another serotype of *Enterovirus* species A, family *Picornaviridae*, which has been involved in sporadic cases or outbreaks documented worldwide since 2008 (2–9), has been one of the major etiological agents associated with HFMD outbreaks in mainland China in recent years (10). Although CVA6 infection generally results in mild and self-limited disease, rarely it can cause severe disease such as aseptic meningitis, encephalitis, and even death (11); therefore, it is very important to understand the molecular characteristics of CVA6.

CVA6 is a nonenveloped positive single-stranded RNA virus with a genome length of about 7.4 kb. The genome is composed of a 5′ untranslated region (UTR), the structural protein P1 region, the functional protein P2 and P3 regions, and a 3′ UTR (12). To date, all CVA6 strains can be classified into four genotypes (A, B, C, or D) based on complete VP1 gene phylogenetic analyses (10). In Qingdao, China, in 2017, a throat swab sample from a 5-year-old girl suspected to have severe HFMD was sent to the Qingdao Municipal Center for Disease Control and Prevention and was identified as CVA6 positive by a commercial real-time reverse transcription-PCR (RT-PCR) test (Liferiver Bio-Tech, Shanghai, China); the throat swab sample from this girl was then transfected into a rhabdomyosarcoma cell line (RD cells) according to standard protocols (13). The infected cells were cultured for 2 passages until cytopathic effect occurred. The supernatant was harvested and stored at −80°C. Viral RNA was extracted with the MagNA Pure LC total nucleic acid isolation kit (product no. REF03038505001; Roche) according to the manufacturer’s recommended protocol. RT-PCR was conducted to amplify the complete genome by using the PrimeScript One Step RT-PCR kit v.2 (product no. RR055A; TaKaRa) (14). The amplified cDNA product was sequenced by BGI (Beijing, China). Primers for PCR amplification and sequencing were designed according to the existing reference sequences (Table 1). Sequences were assembled according to the sequence chromatograms. For the regions not measured, primers for sequencing were designed according to the existing sequences. Finally, the sequences could be spliced into a complete sequence and compared with the reference sequence. Sequencing was performed by Sanger dideoxy sequencing. Sequencing results were assembled with DNASTAR v.6.0 software, and Mega v.7.0 software was utilized to perform sequence alignment and phylogenetic analysis. The phylogenetic tree was constructed with the neighbor-joining method and a Kimura two-parameter model, with 1,000 bootstrap replicates.

**TABLE 1:** Primers used in this study

Oligonucleotide	Sequence (5′ to 3′)	Position (nucleotide range)	Purpose(s)
1CA6F	CGGTTAAAACAGCCTGTGGG	1–17	CVA6 genome sequencing
1268CA6R	CCCGAGCGGTACAAGTAGTG	1249–1268	CVA6 genome sequencing
1113CA6F	TACTCGCCCTGACGTGTC	1113–1130	CVA6 genome sequencing
2059CA6R	GGGAACCAGACCATTGAGTGT	2039–2059	CVA6 genome sequencing
1762CA6F	CTTACAACTGATGACGGGAC	1762–1781	CVA6 genome sequencing
2717CA6R	TTCACCTCCACAACYCCTACYAGC	2694–2717	CVA6 genome sequencing
2333CA6F	TAGACACCCCCACTGAGGCT	2333–2352	CVA6 genome sequencing, CVA6 VP1 sequencing
3530CA6R	GGCAGTAATACACTCCTGTTTGAC	3530–3553	CVA6 genome sequencing, CVA6 VP1 sequencing
3265CA6F	CGCCAAACAATAACTAACACTGC	3265–3287	CVA6 genome sequencing
3714CA6F	GTGATTGGTATCGTGTCCACTG	3714–3735	CVA6 2C sequencing
4579CA6R	GCGGAAGTGAGTACACACTAGAGTG	4555–4579	CVA6 genome sequencing, CVA6 2C sequencing
4441CA6F	CAGTTCAAGAGCAAACACCGTAT	4441–4463	CVA6 genome sequencing
5760CA6R	AGGTACAAACATTGAGGGCATG	5739–5760	CVA6 genome sequencing
5642CA6F	CCCTTGACACTAATGAGAAATTCAG	5642–5666	CVA6 genome sequencing
6921CA6R	AGCAACCATGTTGAGTTCATCC	6921–6942	CVA6 genome sequencing, CVA6 3D sequencing
6493CA6F	GAAGCCAGCAGTTTGAATGACTC	6493–6515	CVA6 genome sequencing
7400CA6R	GTATAACAAATTTACCCCCACCAGT	7400–7424	CVA6 genome sequencing

**Fig 1:**
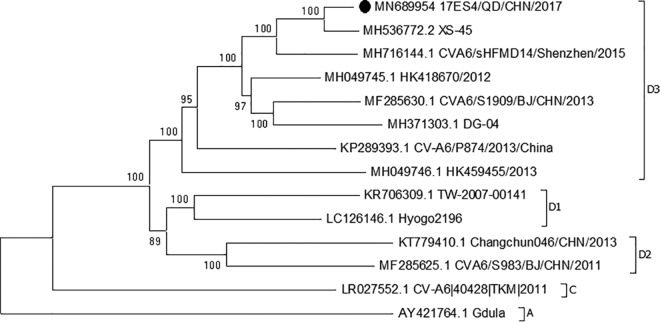
Neighbor-joining phylogenetic analysis of strain 17ES/QD/CHN/2017 (subgenotype D3a). A difference of at least 15% in the entire VP1 region for CVA6 strains was used to distinguish genotypes. Node support was assessed using 1,000 bootstrap replicates. GenBank accession numbers are shown in the tip labels. The black dot indicates the strain sequenced in this study.

The full-length genome of the CVA6 strain 17ES4/QD/CHN/2017 was found to contain 7,435 nucleotides (nt), excluding the poly(A) tail. The 5′ UTR was found to contain 745 nt, followed by a single open reading frame encoding the structural protein P1 (2,610 nt), the nonstructural proteins P2 (1,734 nt) and P3 (2,259 nt), and the 3′ UTR. The isolated CVA6 strain genome has a G+C content of 47.4%. Phylogenetic analyses estimated the viral gene relationships with selected CVA6 strains from GenBank. The results of the phylogenetic analyses show that 17ES4/QD/CHN/2017 belongs to subgenotype D3a, based on phylogenetic analysis of the VP1 gene. Moreover, strain 17ES4/QD/CHN/2017 is closely related to strain XS-45 (GenBank accession no. MH536772.2), with 98% nucleotide identity, based on the results of a complete-genome BLAST search (Fig. 1).

### Data availability.

The full-length sequence of 17ES4/QD/CHN/2017, isolated in Qingdao in 2017, was deposited in GenBank under accession no. MN689954.
